# Monoclonal antibody-based molecular imaging strategies and theranostic opportunities

**DOI:** 10.7150/thno.37443

**Published:** 2020-01-01

**Authors:** Niels Dammes, Dan Peer

**Affiliations:** 1 Laboratory of Precision NanoMedicine, Tel Aviv University, Tel Aviv 69978, Israel; 2 School of Molecular Cell Biology and Biotechnology, George S Wise Faculty of Life Sciences, Tel Aviv University, Tel Aviv 69978, Israel; 3 Department of Materials Sciences and Engineering, Iby and Aladar Fleischman Faculty of Engineering, Tel Aviv University, Tel Aviv 69978, Israel; 4 Center for Nanoscience and Nanotechnology, and Tel Aviv University, Tel Aviv 69978, Israel; 5 Cancer Biology Research Center, Tel Aviv University, Tel Aviv 69978, Israel

**Keywords:** molecular imaging, monoclonal antibodies, autoimmune diseases, oncology and cardiovascular diseases.

## Abstract

Molecular imaging modalities hold great potential as less invasive techniques for diagnosis and management of various diseases. Molecular imaging combines imaging agents with targeting moieties to specifically image diseased sites in the body. Monoclonal antibodies (mAbs) have become increasingly popular as novel therapeutics against a variety of diseases due to their specificity, affinity and serum stability. Because of the same properties, mAbs are also exploited in molecular imaging to target imaging agents such as radionuclides to the cell of interest *in vivo*. Many studies investigated the use of mAb-targeted imaging for a variety of purposes, for instance to monitor disease progression and to predict response to a specific therapeutic agent. Herein, we highlighted the application of mAb-targeted imaging in three different types of pathologies: autoimmune diseases, oncology and cardiovascular diseases. We also described the potential of molecular imaging strategies in theranostics and precision medicine. Due to the nearly infinite repertoire of mAbs, molecular imaging can change the future of modern medicine by revolutionizing diagnostics and response prediction in practically any disease.

## Introduction

On December 22, 1895, the first-ever X-ray picture was taken by Wilhelm Röntgen, who used a Crookes tube to image the hand of his wife, soon after he discovered that Crookes tubes generate a new type of radiation, which he called X-rays 1. Now, 124 years later, modern medicine cannot be imagined without X-rays, which laid the foundation for medical imaging. During these years, improvements such as image enhancement technology, advances in record storing and the development of computer-based image analysis transformed the early X-ray image of 1895 into routine and life-saving applications in modern hospitals 2. Medical imaging nowadays comprises of multiple imaging technologies, some of which rely on ionizing radiation such as X-rays, while some safer alternatives do not employ any ionizing radiation. These imaging techniques are applied to detect a variety of abnormalities such as the presence of cancer, bone fractures or cardiac diseases 3.

Medical imaging is not only used to diagnose a disease, it is also crucial in disease management 4. For example in oncology imaging is a critical tool for assessing tumor shrinkage upon treatment of a patient. Since the mid-20^th^ century, medical imaging was taken one-step further by the addition of probes that facilitate imaging of a specific target site. This resulted in a new discipline called molecular imaging. This new discipline combines traditional medical imaging techniques with novel targeting molecules to visualize the function of various biological processes *in vivo*. Molecular imaging opens new avenues in disease management and personalized treatment as it possesses several advantages compared to traditional medical imaging. One major advantage is the ability to measure response to a treatment at relatively early time points. For example, during cancer treatment, it can take months before anatomical imaging modalities such as CT or MRI are able to measure changes in tumor volume. While still mainly in a pre-clinical stage, molecular imaging has the potential to detect the abundance of specific molecular targets (e.g. VEGFR). Thus, in the near future, it will be feasible to assess therapeutic responses by measuring changes in expression of these molecular targets. This response is detected at an early time point before morphological changes are visible in conventional anatomical imaging. Some other advantages include the ability to measure the molecular marker profile of a tumor in a non-invasive manner thereby eliminating the need for biopsies 5 and the ability to measure bio-distribution of a drug by labeling the drug with an imaging agent 6. The latter is related to a relatively new field called theranostics, which combines treatment with diagnostics to deliver a targeted treatment in a controlled manner and at the same time measure the response with molecular imaging 7.

Medical imaging involves a wide variety of imaging technologies such as positron emission tomography (PET), magnetic resonance imaging (MRI) and ultrasound (US). Table 1 displays a list of common nuclear, optical and other imaging modalities. Each imaging technology has its advantages and disadvantages including, for instance, the ability to provide anatomical information, the use of ionizing radiation, the degree of spatial resolution and the ability to perform whole body imaging. Hybrid imaging techniques are gaining more attention because these techniques can combine imaging modalities that provide anatomical information (e.g. CT) with imaging modalities that provide functional information such as where a target of interest is located (e.g. PET). The functional modality employs a probe that specifically binds to a target such as cancer cells. An overlay between the functional modality and the anatomical modality results in a full anatomical model in which the presence or absence of a signal indicates where the target is located. As molecular imaging relies on suitable imaging probes, this will be discussed in detail in this review with a special emphasis to the use of monoclonal antibodies (mAbs) as the targeting moiety. Three major applications in the field of mAb-based imaging will be discussed: autoimmune disease, oncology and cardiovascular disease.

## Molecular imaging components

### Imaging probes

Imaging probes are the key elements in molecular imaging as they facilitate the emission of a signal specifically from the target site, which is in turn detected by the various imaging modalities. Imaging probes generally consists of two parts: a signal agent (e.g. radionuclide) and a targeting agent (e.g. mAb) that binds to the tissue/cell of interest. While the targeting moiety binds the target, the signal agent produces a signal (e.g. gamma-emitting radionuclides or fluorophores). A linker might be required to bridge these two subunits together. The type of linker can affect the functionality of the targeting moiety and the bio-distribution. Variables in linker design that should be considered include linker length, hydrophobicity, presence of charge and presence of cleavable domains 8. The effect of the linker on imaging probe uptake by the target can be substantial and varies between different types of linkers 9. For instance, Mansour et al. demonstrated that there is a substantial difference in both tumor uptake and non-specific bio distribution between imaging probes linked with an anionic linker and probes linked with a cationic linker 10.

### Imaging agents

Each imaging modality requires a different type of imaging agent. Generally, there are three main types of imaging agents: contrast agents (CT, MRI and PAI), optical probes such as fluorophores (FLECT) and radionuclides (PET, SPECT). Contrasts agents can be targeted to specific sites were they attenuate the imaging signal (e.g. X-ray attenuators are used in CT). Optical agents can include fluorophores for fluorescent imaging or bioluminescent proteins in luminescent imaging. Radionuclides can be targeted to specific tissues and they directly or indirectly emit photons caused by nuclear decay (mechanism depending on the radionuclide used) 11.

Specifically for CT, the use of targeted contrast agents is problematic as large amounts of contrast agents are required to see a change in X-ray attenuation. However, several research groups published a molecular imaging approach using CT by for instance using gold nanoparticles 12 or iodinated nanoparticles 13 as X-ray attenuators. Yet, CT is mostly used in multimodal molecular imaging as an auxiliary technique that provides anatomical information.

Classical MRI imaging uses non-targeted contrast agents such as superparamagnetic iron oxide (SPIO) or paramagnetic gadolinium (Gd) to complement natural tissue contrasts. These contrast agents can be targeted using nanoparticles or monocloncal antibodies and can thereby extend MRI to a functional imaging modality whereas traditionally it is a rather anatomical modality 14.

In PAI, contrast agents need to have a high molar extinction coefficient in order to create sufficient contrast by absorbing light. Ideally, contrast agents absorb in the near-infrared window which is more suitable for imaging in deep tissues. Additonally, the absorbance peak should be a sharp peak to ease spectral unmixing 15. Obvisouly, contrast agents should also possess reactive group for conjugation to targeting moieties. An example is the use of indocyanine green (originally used for fluorescent imaging but it also possesses suitable absorbance properties for PAI 16). This contrast agent was for example targeted to breast cancer using an anti-B7-H3 antibody (a novel breast cancer molecular target) followed by PA imaging 17.

For optical fluorescence imaging popular imaging agents include near-infrared fluorophores such as IRDye 800CW 18, Ag_2_S quantum dots 19 and the FDA-approved indocyanine green 20.

For nuclear imaging techniques such as SPECT and PET, many different radionuclides are available. A key parameter that defines which radionuclide is suitable for a specific approach is the half-life of the radionuclide. As radionuclides decay automatically, they have a specific half-life. Ideally, the half-life of a radionuclide is matched to the half-life type of the probe that should be radiolabeled. Smaller compounds such as peptides have a relatively short serum half-life and can therefore best be conjugated with radionuclides such as ^99m^Tc, ^68^Ga or ^18^F, which have half-lives between 1-6 hours. Larger probes, on the other hand, have longer serum half-lives, especially monoclonal antibodies as they undergo FcRn-mediated recycling resulting in serum half-life times of several weeks 21. Therefore, mAb labeling usually involves different radionuclides such as ^123^I or ^111^In for SPECT imaging and ^64^Cu or ^124^I for PET imaging. The half-life of these radionuclides ranges from half a day to several days 22.

### Antibodies as probes

Antibodies are of particular interest in biomedical research because of their well-defined structure, relative stability, high specificity and high affinity. The remarkable diversity of naturally occurring B-cell receptors is achieved by a complicated process of V(D)J recombination in the developing B-cell's heavy and light chain genes^23^. Subsequent affinity maturation by somatic hypermutation of B-cells in the germinal center of secondary lymphoid organs is the underlying mechanism for the production of B-cells with outstanding affinity towards the specific antigen. During clonal selection in the germinal center, only B-cells with the highest affinity can expand and produce vast amounts of antibodies 24. Advances in *in vitro* affinity maturation enabled researchers to develop antibodies with even higher affinities to their targets making these antibodies desirable in many fields of research 25. Furthermore, as mAbs are mainly produced in mice, these antibodies elicit an immune response resulting in the production of human-anti-mouse-antibodies (HAMAs) which interfere with the treatment 26. Several developments in antibody engineering identified regions that could be 'humanized' without compromising its functionality, resulting in chimeric antibodies and 'humanized' antibodies and this eventually led to the first fully-human antibody produced in transgenic mice, adalimumab 27. All these efforts substantially reduced the immunogenicity of the therapeutic antibodies.

The high affinity and wide repertoire made antibodies of particular interest in targeting strategies such as in the field of molecular imaging. Antibodies are available against a variety of targets such as growth factors, cytokines and cell surface receptors making antibodies useful in molecular imaging in a variety of disease models. An important prerequisite of antibodies is that the target needs to be available extracellularly (e.g. at the outside of cell membranes or as a free molecule in the blood) as targeting of intracellular targets with antibodies is particularly complicated *in vivo* 28. The relative stability and tolerance to chemical modifications are additional benefits of the use of antibodies when creating targeting moieties for radionuclides. Antibodies experience a prolonged circulation time in the body because of their size and structure but more importantly due to their interaction with the neonatal receptor FcRn which protects IgGs against catabolism 29. While this prolonged half-life is desirable in certain applications, in molecular imaging it is sometimes undesirable due to higher background signals and non-specific accumulation in e.g. the liver. Whether this is desirable or not also depends on the half-life of the radionuclide used. How to overcome this and how to modulate antibody half-life is discussed in more detail at the end of this review.

### Conjugation strategies

A major challenge in monoclonal antibody-based imaging is the conjugation of the mAb with the imaging agent. While various techniques can be used, only a fraction of these can be used without compromising the functionality and orientation of the mAb. For radionuclide-based imaging, various chelators are often employed as a linker between the mAb and the metallic radionuclide. This is crucial as the short half-life time of the radionuclide does not allow the time required to conjugate the radionuclide directly to the mAb. Therefore, mAb-chelator conjugates are prepared in advance. This makes metallic radionuclides favorable over non-metallic radionuclides.

While mAbs are relatively stable as compared to other proteins, chemical modification might adversely affect the affinity of the mAb. A major benefit of the use of mAbs in terms of conjugation strategies is the well-defined structure and the presence of functional amino acids for bioconjugation purposes such as thiol groups that are available on the cysteine residues upon reduction of the antibody. We will briefly describe several conjugation strategies that are frequently employed to conjugate the mAb to a suitable chelator.

Examples of chelators include NOTA (1,4,7-triazacyclononane-N,N′,N″-triacetetate), DTPA(diethylenetriamine pentaacetate), DOTA (1,4,7,10-Tetraazacyclododecane-1,4,7,10-tetraacetic acid) and THP (tris(hydroxypyridinone)). In a 2017 study, Moreau et al. compared four different bifunctional chelators in their ability to bind Fab antibody fragments under relatively mild conditions. Their results demonstrated that the chelator MANOTA (Methyl Amino triazacycloNOnane Triacetic Acid) was the most efficient. Their conjugation strategy was based on an isothiocyanate group and involved linkage to the free amine on the lysine residues 30.

Another common approach is the use of carboxyl and amine chemistry. For example, p-NH2-Bn-BFC is used for conjugation to antibodies, in which BFC stands for the bifunctional chelator of choice (e.g. DOTA). This chemical reaction involves the use of EDC (1-ethyl-3-(3-dimethylaminopropyl) as a cross linker which facilitates the covalent linkage of the BFC to the carboxylate residues on the antibody (for instance on Glu and Asp residues)^31^-^33^. A disadvantage is that this procedure requires the antibody solution to be at pH 5, which is not tolerable for all antibodies. Furthermore, the use of primary amines and carboxyl as the reactive group is not very specific as the chelator is attached at any place were the reactive group in the antibody were is present. This might adversely affect the functionality of the antibody.

Click chemistry, a group of reactions that are performed fast and easily by the use of a modular approach 34, enables the site-specific conjugation at physiological conditions (near-neutral pH and ambient temperature). An example of this is based on genetic-code expansion that enables the site-specific insertion of an unnatural amino acid. Oller-Salvia et al., demonstrated in 2018 the incorporation of a cyclopropane derivative of lysine into the antibody trastuzumab in response to amber codons that were created in a recombinant construct 35,36. When using a molecule of interest that contains a tetrazine group, the inverse Diels-Alder reaction allowed the covalent attachment of the molecule of interest to the cyclopropane group in the antibody. In their example they attached monomethyl auristatin E to generate an antibody drug conjugate (ADC), however, tetrazines can be incorporated in common chelators for conjugation purposes 37,38. This enables the covalent attachment of a chelator to the antibody without chemically manipulating the antibody as the reactive group is already added during translation of the mRNA encoding the antibody. We believe that such approaches are superior due to the specificity of the covalent linkage and the lack of chemical manipulation of the protein as the reactive group is added during protein synthesis.

### Use of antibody fragments for decreased background signal

Full-length monoclonal antibodies are popular in research and in medical applications. Antibody production and purification technologies have eased the entire production process and yields increased dramatically. Antibodies are physically stable and experience a prolonged serum half-life due to their interaction with the neonatal Fc receptor, FcRn, resulting in an average half-life of several weeks 29. This long half-life, however, can be unfavorable in imaging applications due to a variety of reasons. The major issue with a too long half-life is the high background signal and too much non-specific tissue accumulation. Large molecules injected *in vivo* do accumulate automatically in certain tissues such as the liver and kidneys. While passive targeting of tumors uses the EPR effect or active targeting to other tissues are designed to minimize the non-specific accumulation in e.g. the liver, residual non-specific accumulation is still unavoidable. The longer the half-life, the more material accumulates non-specifically in other tissues, giving rise to increased background signals that could nullify the target signal. Specifically in PET imaging there is a demand for the use of antibody fragments that are cleared from the circulation more quickly. This is due to the high sensitivity of PET imaging, combined with the high affinity of antibodies; the long circulation time increases the background signal significantly. Furthermore, longer lasting radionuclides are required in PET imaging with full length mAbs which in turn increases radiation exposure in patients.

**Figure 1:**
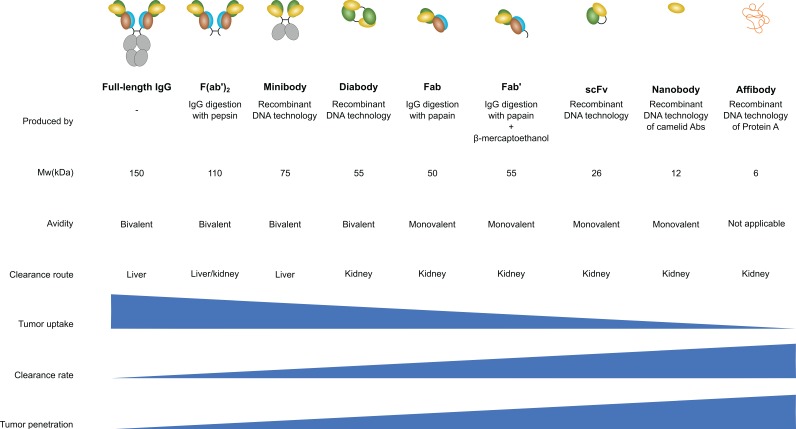
Antibody engineering enabled the production of a wide variety of IgG derivatives. F(ab')_2_, Fab and Fab' products are produced by enzymatic digestion of an IgG molecule while the other derivatives are generated using genetic engineering of IgGs. Nanobodies are specifically engineered from a camelid antibody variant that contains only heavy chains. Figure modified from 41.

Therefore, several antibody-derived products are developed for several different applications. Each antibody-derived product has a different size, bio-distribution and serum half-life. Full-length antibodies can be digested enzymatically either by pepsin, to generate F(ab')_2_ fragments, or by papain to generate fragment antigen-binding (Fab). Another option is to genetically engineer antibodies to generate a variety of products such as scFv or affibody 39. Besides these antibody-derived products some other novel strategies are devised where (parts of) antibodies are fused to domains of other proteins (the chimeric antigen receptor, for instance, is a scFv that is fused to a signaling domain such as CD3ζ 40). Figure 1 shows the different antibody-derived products, their size, *in vivo* kinetics and clearance mechanism (renal vs. liver).

Examples of applications of antibody-derived products in molecular imaging include the use of a scFv against the ion channel hERG1 for cancer optical imaging 42, the use of a minibody against PSCA using PET 43 and the use of a PSCA-targeted diabody in a PET/optical imaging hybrid^44^. In all these studies, the incentive to use antibody fragments was mainly due to the faster clearance from the circulation.

The smallest antibody derivative is the affibody, which consists of merely 58 amino acids residues that form three helix bundles. Affibodies combine high affinity with rapid uptake and quick clearance which make them useful for PET imaging by creating a high contrast. For instance, a recent study in 2019 reported the use of an affibody against HER2 in PET imaging 45. Although a bit larger, nanobodies are also popular due to their fast clearance. Like affibodies they possess a relatively high chemical and temperature resistance due to their small size and less complex 3D structure. Obviously, this is favorable for molecular imaging procedures as this opens more possibilities for conjugation chemistry to chelators, contrast agents or optical probes 46. An example of the use of nanobodies in molecular imaging is a study that integrated the targeting of three different targets in a multimodal fashion using both PET and MRI to detect atherosclerotic plaques 47. This example is also addressed in the atherosclerosis section.

While most studies that utilize antibody's fragments emphasize the superiority over IgGs by their faster clearance while maintaining similar binding affinities, the benefits of high binding affinities are also being questioned. Fujimori and colleagues, back in 1990, suggested that in tumor imaging the targeting moiety with the highest affinity might not be the best choice 48. They hypothesize that a too high binding affinity prevents penetration into the tumor due to an effect they refer to as the binding site barrier. This barrier limits tumor penetration because of excessive binding to the receptors at the tumor surface. This prevents tumor penetration and leads to heterogeneity of intra-tumor distribution. Furthermore, a higher affinity does not always correlate with increases tumor retention. No improvement in tumor retention is observed beyond a specific optimum. This optimum, however, depends on the size of the targeting protein. Smaller targeting proteins such as affibodies require higher affinities to reach the tumor retention optimum 49.

We believe that it is clear that in nuclear imaging, smaller antibody fragments such as affibodies are probably going to replace the full-length IgG molecules. In other imaging modalities, it still needs to be seen whether full-length IgGs or derivatives are more suitable. Some emerging optical imaging technologies such as FLECT might be well suited to IgGs while for PET imaging affibodies could well be the new standard. Overall, achieving high contrast, homogenous, fast and reproducible imaging data with high sensitivity and resolution is a complex interplay between targeting proteins and imaging agents. While most labs focus on achieving the highest possible affinity, the binding site barrier theory suggests that proteins with a lower affinity might be better suited for homogenous tumor imaging.

## Applications of molecular imaging

### Autoimmune diseases

Autoimmune diseases affect around 3-5% of the general population. There are many types of autoimmune diseases such as Crohn's disease, multiple sclerosis and Graves' disease. Generally, autoimmune diseases are caused by an overactive immune system and a lack of immunological tolerance 70. During a normal immune response, the immune system reacts very quickly to infections while at the same time, almost immediately after the immune response started, signals that inhibit the immune system are released 71. This tight control ensures that the inflammatory response ends early enough to prevent unnecessary injury. Generally, this process is insufficient in patients suffering from autoimmune disorders but the underlying cause varies greatly between the different diseases and between individual patients. As many autoimmune diseases are heterogeneous, it is hard to predict if an individual patient's disease will relapse or not. Furthermore, diagnosis of inflammation relies on classical signs of inflammation (swelling, pain, redness and heat) and these signs may appear at a later stage of the disease, where irreversible tissue damage could already have been taken place 71. A wide repertoire of drugs are available against the various autoimmune diseases ranging from classical immunomodulatory drugs such as corticosteroids to novel biologics blocking key cytokines such as TNF-α. However, when diagnosed too late, even the most promising drugs might not be able to reverse the disease or recover the damaged tissue.

Disease biomarkers can be detected in the urine or blood but the most conclusive way of diagnosis relies on biopsies. Biopsies are analyzed by either light microscopy, immunofluorescence microscopy or electron microscopy 71. Immunofluorescence microscopy can detect crucial indicators of autoimmune disorders like the upregulation of various cell adhesion molecules in the vasculature (required for leukocyte homing) 72,73. Molecular imaging, however, can provide a non-invasive alternative to detecting such biomarkers. This helps the clinicians to decide whether to start, continue or stop therapy. These choices are of utmost importance in patients suffering from autoimmune disorders as these diseases have a chronic nature and clinicians continuously need to decide whether to repeat therapy or not.

Therapeutic antibodies against the important pro-inflammatory cytokine TNF-α were approved already in the 1990s for use in rheumatoid arthritis (RA). A study from 2005 demonstrated successful radiolabeling of a chimeric anti TNF-α antibody, infliximab, by reducing the disulfide bonds and conjugating the reduced antibody with methylene diphosphonate which complexes with radioactive technetium, ⁹⁹ᵐTc. Using scintigraphy, the authors could accurately image inflamed joints using the ^99m^Tc-infliximab while non-inflamed joints did not show any uptake. More importantly, after treatment with unlabeled infliximab the uptake of ^99m^Tc-infliximab decreased, indicating that the treatment reduced inflammation and TNF-α expression resulting in less available targets for ^99m^Tc-infliximab 74. A study back in 2003 demonstrated similar results using a fully human antibody against TNF- α, adalimumab. The Ab was radiolabeled indirectly using succinimidyl-hydrazino nicotinamide (S-HYNIC), which is a bifunctional chelator and can capture radioactive technetium 75. The ^99m^Tc-adalimumab specifically labeled inflamed joints while ^99m^Tc-adalimumab uptake in non-inflamed joints was absent. To test the dependency on TNF-α expression, the researchers did a competition experiment by co-administrating excess unlabeled adalimumab. This decreased the signal in inflamed joints by 25%, indicating the dependency on the presence of TNF- α in the affected joints. Treatment with corticosteroids, which are known to reduce the inflammation, resulted in lower ^99m^Tc-adalimumab uptake, proving the suitability of this approach in the assessment of therapy response. The ^99m^Tc-adalimumab uptake was not evident in all inflamed joints, most likely because not all inflamed joints are characterized by TNF-α expression, this is important for pre-selection of patients for anti-TNF-α therapy. A mini review compared studies using both ^99m^Tc-infliximab and ^99m^Tc-adalimumab and concluded that both antibodies are suitable for therapy decision-making purposes in RA 76. Another interesting observation in that study was that patients that showed high pre-therapy uptake of ^99m^Tc-adalimumab showed more therapeutic benefit when treated with unlabeled anti-TNF mAb 76,77.

Another study explored the possibility of using E-selectin as a target. E-selectin is an adhesion molecule that is expressed on vascular endothelium. It is overexpressed by pro-inflammatory signals such as IL-1 during inflammatory disorders. Keelan et al. published in 1994 an animal study in which an anti-E-selectin antibody (clone 1.2B6) was radiolabeled with ^111^In. The labeled antibody was injected intravenously in pigs which were subsequently analyzed by scintigraphy. The results showed specific accumulation of the labeled antibody in the inflamed knee and this was significantly higher than a labeled isotype control antibody 78. A follow-up study compared two fragments of the 1.2B6 antibody against E-selectin in terms of diagnostic accuracy using scintigraphy. ^99m^Tc-labeled 1.2B6 Fab was compared with ^111^In‐labeled 1.2B6 F(ab′)2 in human patients. The ^111^In-labeled F(ab')2 fragment of 1.2B6 already turned out useful in RA diagnostics as shown previously^79^ and the authors wanted to test the suitability of ^99m^Tc because ^111^In is an expensive radionuclide and it exposes the patient to relatively high radiation doses. A Fab fragment of 1.2B6 was chosen to test the suitability of ^99m^Tc due to its fast blood clearance, which limits the signal from non-target tissues. The ^99m^Tc-labeled 1.2B6 Fab turned out as effective as the ^111^In‐labeled 1.2B6 F(ab′)2 and both methods were superior to conventional bone scanning techniques^80^. This study represents a promising step forward in disease management of autoimmune diseases as E-selectin is rarely expressed in healthy tissues while it is overexpressed on the endothelium of inflamed tissues.

As leukocytes are a central part of the immune system and as they are indispensably involved in the inflammation, targeting leukocytes could accurately image autoimmune disorders. Several strategies were devised to accomplish this by targeting radionuclides to either CD3^+^, CD4^+^ and CD20^+^ cells. The murine mAb OKT-3 against CD3 was employed several times in human studies to test the effectiveness in disease management. Marcus et al. showed that ^99m^Tc-anti-CD3 mAb can be used to assess therapeutic effectiveness in RA patients. All painful joints demonstrated ^99m^Tc-anti-CD3 mAb uptake, however, some unexpected side effects occurred (most likely cytokine release syndrome related) limiting its future applicability 81. This could be due to the immunogenicity of the murine antibody as it is well-known that humans produce HAMAs (see molecular imaging components section). However, in 2008 Martins et al. published a study with the same goal and the same antibody but with a different radiolabeling strategy (Marcus et al. reduced the antibody with ascorbic acid while Martins et al. used 2-mercaptoethanol). Side effects were absent in this study, underscoring the importance of choosing the right radiolabeling strategy for each application 82. Becker et al. showed that besides targeting the entire T-cell population, a radiolabeled monoclonal antibody against CD4 can label specifically T-helper cells and provides an early and accurate diagnosis of inflamed joints using scintigraphy 83. Another strategy targeting B-cells using an anti-CD20 mAb, rituximab, radiolabeled with ^99m^Tc was shown to be successful by Malviya et al. The researchers demonstrated uptake of the radionuclide by B-cells in a variety of autoimmune diseases, proving the universal nature of this approach. Over time, the role of B-cells in mediating chronic inflammation in autoimmune disorders became more apparent. The role of B-cells in these processes range from the production of auto-antibodies to T-cell activation and pro-inflammatory cytokine production 84.

**Figure 2:**
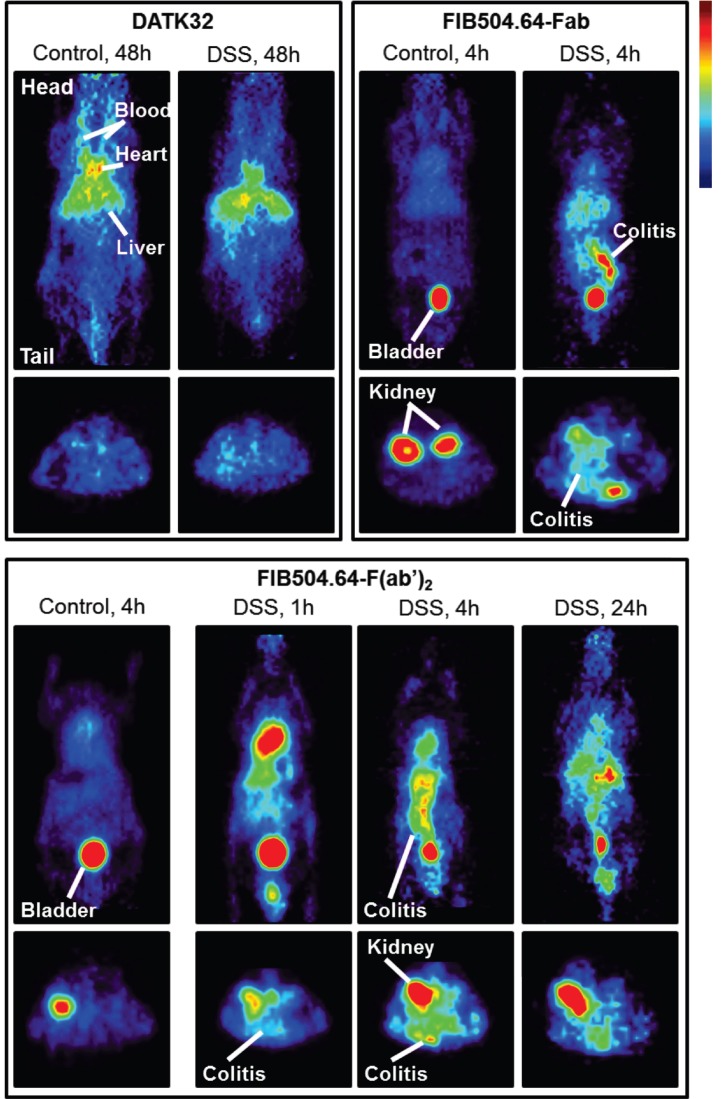
Imaging data of ^64^Cu-labeled anti-α_4_β_7_ integrin (DATK32) and anti-β_7_ integrin (FIB504.64), the latter with either Fab or F(ab')_2_ antibody fragments. Coronal images are shown in the upper panel and transaxial images in the lower panel. The FIB504.64 clearly shows a better, more specific uptake than the DATK32 antibody. The presence of colitis is evident in the images when compared to healthy control mice, indicating the specificity. This figure is reprinted with permission from Inflammatory Bowel Diseases (Dearling et al., 2016) 33.

In 2010 and 2016, Dearling et al. published two studies in collaboration with our group to image colitis, an autoimmune disorder involving the gastrointestinal tract. These studies employed ^64^Cu to image the disease using microPET imaging. As the homing receptor α_4_β_7_ integrin is a crucial receptor of leukocytes homing to the gut, the first study utilized an antibody against β_7_ integrin. This rat mAb, FIB504.64, against human and mouse β_7_ integrin was conjugated to the bifunctional chelator DOTA. After inducing experimental colitis in C57BL/6 mice using dextran sodium sulfate (DSS), FIB504.64-DOTA was labeled with ^64^Cu and injected intravenously. At 48 hours post injection, the mice were imaged with a microPET imaging device. The researchers were able to detect a radioactive signal in the inflamed gut while the level of radioactivity was less profound in healthy mice 32, see Figure 2. However, significant levels of non-specific uptake were also evident which led to the second study in 2016 that tried to overcome this problem. The second study utilized an antibody against α_4_β_7_ integrin named DATK32 and the F(ab')_2_ and Fab fragment of the FIB504.64 mAb. The rationale was that the DATK32 should provide more specific targeting while the F(ab')_2_ and Fab fragments of FIB504.64 should be cleared from the blood more quickly thereby eliminating unspecific uptake. The FIB504.64- F(ab')_2_ fragment demonstrated the highest differential between mice with colitis and healthy mice and was therefore the best candidate for potential future imaging studies of colitis. The DATK32 mAb showed lower intestinal uptake and targets probably a too specific subpopulation of leukocytes for *in vivo* imaging purposes 33.

Although the initiation of DSS colitis is mainly driven by neutrophils and macrophages 85, later studies showed that DSS triggers the influx of lymphocytes to the gut and these lymphocytes exacerbate the disease 86. This inspired Kanwar et al. to image CD4^+^ T-cells in the gut with a radiolabeled anti-CD4 antibody (^111^In-DOTA-anti-CD4 mAb). Despite the fact that they demonstrated that the CD4^+^ cells only represent a small fraction of the total lymphocyte population, they were able to image the CD4^+^ cells in the gut specifically in mice with colitis using SPECT/CT^87^. The problem of their approach, however, is that the CD4^+^ cells can represent both helper T cells and regulatory T cells. By only targeting CD4, we believe that one cannot conclude whether the cells that have taken up the radionuclide are pro- or anti-inflammatory.

While Kanwar et al. injected 350 µg of radiolabeled-antibody; in 2018 Freise et al. reported similar results with doses as low as 2 µg. Flow cytometry results revealed that the percentages of CD4+ cells in the mesenteric lymph nodes (MLN) were lower in DSS colitis mice than in healthy controls. However, as the MLN in mice with colitis were significantly increased, the total cell number was much higher than in healthy controls. Therefore, the uptake of radiolabeled antibody in the MLN was higher than in healthy controls as was shown in the imaging data 88. Still, the CD4-targeted radionuclide uptake cannot conclude whether these CD4^+^ cells possess an inflammatory phenotype or not.

### Oncology

In contrast to antibodies targeting cytokines or leukocytes, which are bound in the bloodstream, targeting solid tumors depends greatly on the diffusion of the antibodies from the vasculature into the tumor. The enhanced permeability and retention (EPR) effect has been thoroughly investigated, being a unique property of tumors that enhance diffusion of macromolecules through the leaky vasculature in the tumor area. Due to the rapid growth of tumors, the surrounding blood vessels have a defective architecture and in some cases produce various permeability factors to ensure sufficient influx of oxygen and nutrients 89. This feature is exploited in cancer therapy to enhance tumor accumulation of macromolecules. The molecules first pass the endothelial barrier followed by movement through the tumor interstitium and extracellular matrix before they reach the tumor cells. Generally, compounds greater than 40 kDa benefit from the EPR effect and can accumulate at the tumor site. An additional benefit of these macromolecules is their prolonged circulation time compared to smaller molecules that are rapidly cleared by the liver and kidneys 49. While targeted molecules have tumor targeting abilities, their initial diffusion from the vasculature into the tumor tissue relies on the EPR effect like untargeted molecules. Due to the inter- and intra-patient heterogeneity of the EPR effect, treatment efficacy varies between patients, leading to dissimilar outcomes in clinical trials. The EPR effect is so heterologous because multiple vascular as well as micro-environmental factors contribute to the EPR. At the vascular level, main contributors are vascular permeability, expression of endothelial receptors and the degree of vascular maturation (as tumors rapidly enhance angiogenesis the maturation state is significantly different from healthy tissues). Micro-environmental contributors include the extracellular matrix, presence of hypoxia, interstitial fluid pressure and tumor cell density 90. When taking into account all these different contributors, one can imagine the complexity and understand the heterogeneous nature. Because of this EPR heterogeneity, antibodies provide an opportunity for active targeting that can enhance the passive targeting of the EPR effect. Examples of enhancing tumor targeting include antibody-drug conjugates (APCs) which consist of a drug (e.g. chemotherapy) linked to a mAb that drives the specific delivery to the tumor. Examples of ADCs include Gemtuzumab-ozogamycin (targeting CD33) 91, Brentuximab-vedotin (targeting CD30) 92 and Trastuzumab-emtansine (targeting HER2) 93. Besides merely functioning as a targeting moiety, the therapeutic potential of mAbs themselves have been appreciated as naked antibodies demonstrated profound therapeutic efficacy 94. mAbs can inhibit tumor growth by several mechanisms: (i) activation of antibody-dependent cellular cytotoxicity (ADCC) driven by Fc-recognizing cells such as NK cells, (ii) tumor growth inhibition by blocking important cellular receptors with mAbs (e.g. blocking EGFR or HER2/neu) and (iii) activation of the immune system by blocking immune inhibitory receptors with mAbs (e.g. immune checkpoint inhibition therapy) 94.

Besides using mAbs for therapy, their long circulation time makes mAbs excellent targeting molecules for molecular imaging modalities (although the effective circulation time also depends on the stability of the radionuclide used). Several strategies regarding molecular imaging of tumors will be briefly discussed in the rest of this chapter.

Human epidermal growth factor 2 (HER2) was identified in the 1980s as an oncogene in breast cancer 95 and since then, it has been a focal point of research in the past decades. This resulted in an FDA approved antibody, trastuzumab that blocks HER2 signaling. Treatment of breast cancer patients with this monoclonal antibody positively affected the prognosis due to the important role of HER2 in proliferation and metastasis of cancer cells 96. This therapy, however, only has an effect in women that have breast cancer that is positive for HER2 and this only accounts for ~20% of breast cancer patients^97^. This makes it important to screen patients who are HER2 positive and this is generally done using immunohistochemistry analysis of the primary tumor. However, as HER2 expression can change during the course of the disease and can be unequally expressed across tumor lesions or metastases, a non-invasive continuous assessment of HER2 expression is demanded. Therefore, substantial effort was conducted in the scientific community to generate molecular imaging strategies to assess HER2 expression. A review article from 2010 summed up the tremendous efforts that were put into the generation of many different types of HER2 antibody/antibody fragment imaging modalities 98. Several studies developed formulations of radiolabeled trastuzumab such as ^111^In-DTPA-trastuzumab 99, ^188^Re (I)-trastuzumab 100, ^111^In-DTPA-trastuzumab-IRDye800 101 or ^64^Cu-DOTA-trastuzumab-IRDye800 102 (the latter two represent dual-labeling strategies combining radionuclide imaging with fluorescent imaging). This resulted in multiple in-man imaging studies using trastuzumab to image HER2 expression. We found seven studies that used trastuzumab in humans (updated until 2018). Four of these studies utilized ^111^In 55,103,104,105, two studies ^89^Zr 106,107 and one study ^64^Cu 108. Although the patient sizes in these studies were relatively small, this represents an encouraging step towards the routine use of molecular imaging in patients to accurately assess HER2 expression over time. A publication by Massicano et al. was entirely devoted to the targeting of HER2 using molecular imaging 109. Numerous preclinical studies are listed in this paper utilizing different radionuclides and different targeting moieties (also beyond the use of trastuzumab). This highlights the urgency for developing less invasive methods to monitor HER2 expression over time. We believe that the variety of approaches that are currently in a preclinical pipeline will likely yield at least one promising candidate that could be used clinically in the future and will replace classical methods of HER2 evaluation. Molecular imaging has the advantage of being able to quantify HER2 expression accurately at every location in the body.

Two other growth factor related proteins that have been intensely investigated in oncology are EGFR and VEGF. For both targets, FDA-approved monoclonal antibodies are available for cancer treatment and nowadays these antibodies are also tested in their predictive abilities when used in molecular imaging. For instance, Nagengast et al. demonstrated how radiolabeled bevacizumab can target VEGF in the tumor area of an ovarian tumor xenograft 110. Furthermore, Hoeben et al. published an animal study in 2010 where they tested the tumor uptake of radiolabeled cetuximab (anti-EGFR). The study reported tumor uptake and calibrated the imaging modality with several doses and time points. Moreover, the residualizing properties of the radionuclides were also taken into account and to this end; the researchers compared ^111^In‐cetuximab (residualizing) to ^125^I‐cetuximab (non-residualizing). Inidum-111 turned out more successful in terms of tumor uptake in all time points, probably due to the internalizing properties of cetixumab 111. As cetuximab internalizes, a residualizing radionuclide seems better fitted as they stay in the lysosome and therefore remain in the cell for a longer period. Non-residualizing radionuclides easily end up in the extracellular space as a free molecule while the antibody is degraded 112. Zirconium-89, another residualizing radionuclide was conjugated to cetuximab and investigated in two clinical trials for its predictive power in cancer patients 113,114. The clinical trial IDs are: NCT00691548 and NCT01504815 (the latter trial is still ongoing). Despite the potential of theranostic modalities, in certain cases treatment and diagnosis cannot be carried out simultaneously. In the case of cetuximab (anti-EGFR) for instance, treatment and diagnosis have been shown to interfere with each other because of competition between the therapeutic antibody and the cetuximab-radionuclide conjugate (as they recognize the same epitope in EGFR domain III) 111. As all approved mAbs against EGFR recognize specifically domain III, development of other antibodies that recognize different epitopes are demanded. Furthermore, specific EGFR mutations that occur in tumors make the EGFR resistant to antibodies. As these mutations occur in domain III, the tumors cannot be accurately diagnosed with the currently approved antibodies. An exception to this is the domain III binder panitumumab which recognizes the same epitope as cetuximab but remarkably can overcome cetuximab-induced resistance due to a slightly different binding mechanism 115. Aghevlian et al. used this antibody as a theranostic modality by targeting EGFR in pancreatic cancer 116. In that study, they showed that the use of a metal-chelating polymer that harbors multiple DOTA residues has a higher labeling efficienc than DOTA-conjugated panitumumab. Bernhard et al. developed IRDye800CW-labeled scFvs that recognize EGFR domain I/II and they demonstrated that co-treatment with a domain III-binding antibody does not interfere with binding efficiency of the imaging probe. Furthermore, they used these labeled scFvs together with the conventional domain III binder nimotuzumab to assess the ratio of WT and mutant EGFRvIII, a mutation that lacks domain I and most of domain II (common in glioblastoma) 117. If more antibodies that recognize different EGFR domains are available, phycisians will be able to assess the abundance of certain EGFR mutations and can treat the patients with the correct antibodies. These will most likely increase the amount of responders and it will enable the simultaneous treatment and diagnosis without interference.

A study from 2018 by Tsai et al. 118 created a dual-imaging modality based on a minibody (an 80 kDa recombinant product of a mAb) that was conjugated to both a radionuclide and a fluorophore. The minibody recognized PSCA, a protein that is up regulated in many prostate cancers. While the radionuclide is useful for diagnosis, the fluorophore can be used in near-infrared fluorescence (NIRF) image-guided surgery, so the researchers generated one product that can be used both in diagnosis and during surgery. While it is in principle a great idea to combine both imaging probes in one formulation, we doubt the practicality of this approach. In case the imaging probe is already cleared from the body by the time surgery takes place (after initial diagnosis aided by the imaging probe), a new dose should be administered to aid during surgery. However, at that time the radionuclide is not necessary anymore as PET images have already been taken. In such a scenario, a dual-imaging modality will just expose the patient unnecessarily to extra radioactive molecules while only the fluorophore is required. In case surgery takes place the same day or the day after diagnosis, this approach has more potential.

**Figure 3:**
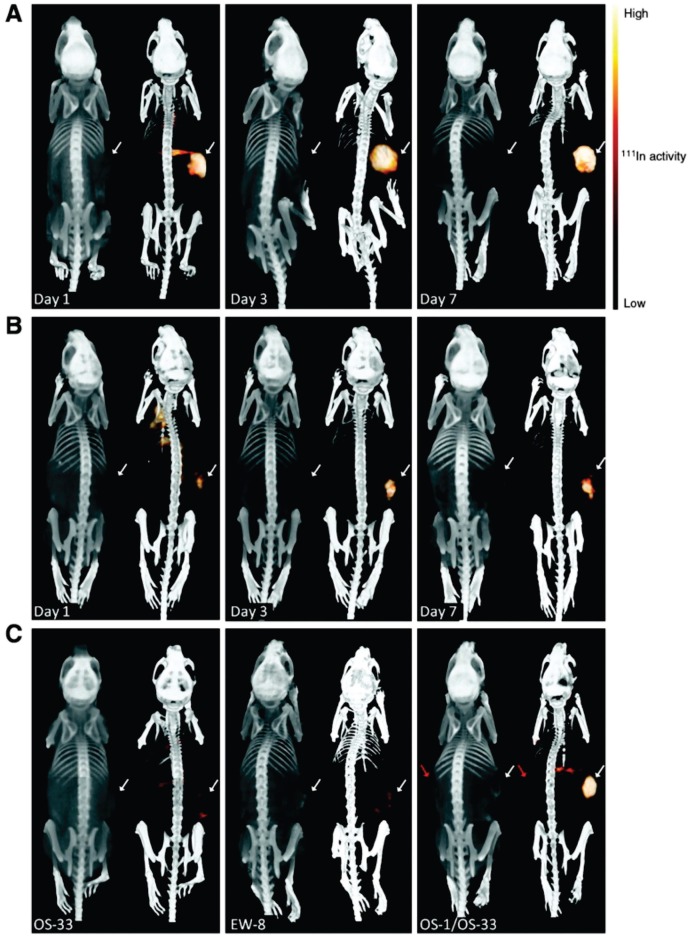
Bio-distribution of ^111^In-R1507 (against IGF-1R) in three different mouse models of bone sarcoma. **A** are mice with IGF-1R positive tumors that did respond to anti-IGF-1R therapy. **B** are mice with IGF-1R positive tumors that did respond only modestly to anti-IGF-1R therapy. **C** shows the three different models side-by-side (all imaged at day 3). OS-33 is IGF-1R negative, EW-8 is IGF-1R positive but does not respond to anti-IGF-1R therapy and OS-1 is IGF-1R positive and shows a response to anti-IGF-1R therapy. The mouse in the right panel has two tumors, OS-1 (indicated with the white arrow) and OS-33 (indicated with the red arrow). As can be derived from the figure, only OS-1, a IGF-1R positive tumor that responds to the treatment shows significant uptake of ^111^In-R1507. This adapted figure is reprinted with permission from Clinical Cancer Research (Fleuren et al., 2011) 121.

In 2011, Fleuren et al. published a study based on the previsouly formulated ^111^In-R1507 119, a radiolabeled antibody against insulin-like growth factor 1 receptor (IGF-1R), and tested if ^111^In-R1507 is a predictive marker for response by bone sarcoma patients to anti-IGF-1R therapy. Their results demonstrated that mice with tumors positive for IGF-1R that did not respond to anti-IGF-1R therapy showed ^111^In-R1507 uptake similar to mice harboring IGF-1R negative tumors and only mice positive for IGF-1R that responded to anti-IGF-1R therapy had a significant uptake of ^111^In-R1507 (see Fig 3).

Another novel target for molecular imaging in cancer are activated platelets. As activated platelets tend to accumulate in tumors, imaging platelets might provide a more universal approach than targeting specific receptors. A study in 2017 demonstrated the feasibility of targeting activated platelets using a scFv against the activated integrin GPIIb/IIIa. The scFv was conjugated to Cy7, ^64^Cu and ultrasound-enhancing microbubbles. This way fluorescence imaging, PET imaging and ultrasound were compared in terms of feasibility^120^. This study demonstrated a proof-of-concept for imaging activated platelet accumulation in tumors and opened a new avenue in universal imaging modalities for diagnosis of cancer.

In the field of theranostics, the aim is to have a dual modality that enables both treatment and diagnosis/disease management. This is particularly important in the emerging field of immunotherapy. Despite the spectacular achievement in cancer immunotherapy, only a small subset of patients responds 122. Therefore, attention is now shifting to the development of novel approaches that accurately predict whether a patient responds or not and to the development of treatments, that can turn the 'cold', non-responding, tumors into 'hot' tumors that respond to cancer immunotherapy. Furthermore, the scope of cancer targeting is moving beyond merely targeting tumor cells. As there is mounting evidence showing the importance of the tumor immune microenvironment, targeting components of this microenvironment is prompting more attention. The tumor immune microenvironment drives immune suppression and thereby drives tumor progression. The immune suppression is facilitated by both immune suppressive cells such as tumor associated macrophages (TAMs) and myeloid derived suppressor cells (MDSCs) as well as inhibitors such as TGF-β and indoleamine 2,3-dioxygenase (IDO). These immune suppressors inhibit the infiltration and proliferation of e.g. cytotoxic T lymphocytes (CTLs) and thereby promote tumor progression 123.

Theranostic modalities can have three different anti-cancer strategies: by targeting the tumor itself, by targeting the tumor immune microenvironment and by targeting the peripheral immune system to activate immune cells that invade the tumor 123. Molecular imaging has the ability to assist in all three strategies by accurately assessing whether or not a treatment is or will be effective. An example of a theranostic approach to activate the peripheral immune system is a reported pre-clinical study that radiolabeled an anti-PD-L1 antibody, MX001, in order to monitor tumor PD-L1 expression using nuclear imaging during anti-PD-L1 treatment 124. We believe that such the integrative approach of combining treatment with disease monitoring is going to be the main area of impact that molecular imaging techniques in oncology will have.

### Cardiovascular diseases

Cardiovascular disease (CVD) is a leading cause of mortality and hospitalization globally. The main cause of CVD is atherosclerosis leading to for instance myocardial infarction, stroke and heart failure. Atherosclerosis is a chronic disease characterized by the accumulation of lipids and fibrous elements in the main arteries leading to plaque formation and lesions. While advanced lesions can grow gradually to eventually block the blood flow, most clinical complications are the results of acute occlusions as a result of plaque rupture. The disease is divided in multiple stages, starting with lipoprotein entrapment in the sub endothelial matrix, resulting in lipoprotein aggregation, which ultimately forms foam-cells (a form of macrophages) and fibrous lesions 125. An important parameter in atherosclerosis is plaque stability. Unstable plaques can rupture and lead to acute coronary syndromes. As inflammation is ongoing in fibrous plaques, immune cells play an important role in the formation of unstable plaques. Atherosclerosis is considered an asymptotic disease that slowly develops over a period of many years until plaque rupture results in acute clinical symptoms, therefore, plaque stabilization is proposed as a possible therapeutic intervention 126.

To assess the risk of an individual for the development of CVD, accurate assessment of plaque stability is crucial. Molecular imaging enables the non-invasive assessment of atherosclerotic plaques' stability and can thereby determine the severity of the disease. A study back in 2004 described how oxidation-specific antibodies labeled with ^125^I can detect lipid-rich, oxidation-rich plaques. This is important, as the presence of highly oxidized LDL in plaques is associated with plaque vulnerability 127. The study demonstrated the presence of highly oxidized LDL using the MDA2 antibody, which binds malondialdehyde-lysine epitopes. The antibody recognized atherosclerotic lesions while avoiding normal arteries in both mice and rabbits. The radiolabeled antibody turned out highly sensitive to atherosclerosis regression due to a loss of oxidation-specific epitopes. Therefore, a decrease in oxidized LDL directly resulted in a decreased radioactive signal. The research suggested that serial imaging by intravenous injection of ^125^I-MDA2 can detect changes in the content of oxidized LDL in plaques and therefore predict plaque stability 128. This opens new venues in disease management in people who are at high risk for atherosclerosis. A follow-up study in 2013 by Briley-Sabeo et al. 129 also used an antibody against MDA2 that was labeled with manganese to facilitate manganese-enhanced magnetic resonance imaging (MRI). Manganese (Mn) was suggested as a safer alternative to gadolinium (Gd), the most commonly used MRI contrast agent. Micelles labeled with Mn and micelles labeled with Gd were targeted with an antibody against MDA2. Both formulations were tested in two different mice models and the results showed that Mn was at least as effective as Gd. Because Mn has a poor MRI efficiency when chelated, this method relied on the intracellular delivery of Mn as it is released from the chelator upon cell entry. The authors showed intracellular delivery to foam-cells, cells that are highly predictive for monitoring high-risk atherosclerosis. Similar to the 2004 study, this study showed that targeting oxidation-specific epitopes is highly predictive for assessing plaque stability and atherosclerosis severity. The researchers suggested that manganese-enhanced MRI is a safe and biocompatible method that can efficiently be applied to detect oxidation-specific epitopes in atherosclerosis.

Ultrasmall superparamagnetic iron oxide (USPIO) nanoparticles are a novel class of contrast agents in MRI. A study by Wen et al.^130^ in 2014 demonstrated how targeted USPIO nanoparticles accurately detect atherosclerotic lesions in an ApoE-deficient mouse model of atherosclerosis. The researchers used PEG-coated USPIO and conjugated these to an anti-LOX-1 antibody. LOX-1 (expressed in atherosclerotic plaques by smooth muscle cells and macrophages) has been shown to play an important role in destabilization of plaques, meaning that LOX-1 expression in plaques is positively correlated with plaque instability 131. The study indeed demonstrated specific imaging of LOX-1 in atherosclerotic lesions proving that the LOX-1-targeted USPIO nanoparticles have clinical potential in imaging unstable plaques in atherosclerosis patients.

As the immune system plays a causal role in plaque rupture, other imaging approaches focus on determining the expression levels of endothelial cell adhesion molecules (ECAMs) such as ICAM-1 VCAM-1 or selectins at plaque vulnerable regions to predict the influx of leukocytes. For example, a study in 2010 demonstrated the detection of lesion-prone vascular sites in the early pathogenesis of atherosclerosis. The researchers could detect vascular abnormalities before the development of fatty streaks when monocytes just start to accumulate at the affected site. This study utilized contrast-enhanced ultrasound, which employs microbubbles that accumulate at sites rich in ECAMs. The microbubble accumulation in ECAM-rich sites was achieved by the conjugation of the microbubbles with monoclonal antibodies against P-selectin and VCAM-1^132^. The microbubbles therefore detect sites of atherosclerosis pathogenesis before lesions occur. This opens possibilities in early risk stratification of atherosclerosis for the future.

Because atherosclerosis is a chronic, latent disease, it is beneficial to diagnose patients at an early stage to prevent severe symptoms that occur during acute injury at a later stage. However, once an acute event eventually takes place, a rapid diagnosis is required to prevent as much damage as possible. Therefore, Davidson et al. 133 developed a similar method using contrast-enhanced ultrasound in 2012 but they applied it for the rapid detection of acute myocardial ischemia. Multi-selectin-targeted microbubbles were employed as contrast agents and yielded promising results in mice. Another ultrasound-based method employed a triple targeting system by integrating an antibody against VCAM-1, antibody against ICAM-1 and a synthetic polymer of the saccharide sialyl Lewis X. The researchers attempted to mimic leukocyte recruitment to the blood vessel wall at the start of atherosclerosis and demonstrated that this approach serves as a suitable imaging probe for early diagnosis of atherosclerosis 134.

In the field of cardiovascular diseases, targets of interest are P-selectin, αVβ3 integrin and fibrin. There is a strong relation between elevated P-selectin levels and the development of thrombosis 135 and therefore P-selectin was thoroughly investigated in the context of diagnosing thrombosis 136,137. αVβ3 integrin assessment is important to gain insights in cardiac repair as this integrin is a key mediator of the repair process and its expression is increased at sites of recent myocardial infarction. Jenkins et al. described the employment of PET and CT to image sites of post myocardial infarction repair using ^18^F- Fluciclatide which targets αVβ3 integrin (clinical trial identifier NCT01813045). Fibrin is of particular interest for detecting thrombosis as it is highly concentrated in the thrombus and it plays an important role in thrombus formation. Fibrin can be targeted by peptides as this was demonstrated in several studies that employed PET, SPECT and several optical modalities 138. Furthermore, fibrin targeting assists in the development of an accurate diagnosis of stent thrombosis, a potentially lethal complication of coronary artery stents. Fibrin deposition is a characteristic of unhealed stents and targeting of fibrin by near-infrared fluorescence enhances detection and diagnosis of this complication 139.

A major milestone was achieved with the development of ^18^F-GP1, a novel PET probe that targets glycoprotein IIb/IIIa receptors 140. A hallmark of a developing thrombus is the deposition of activated platelets with increased expression of glycoprotein IIb/IIIa. Recently, a publication was released 141 that described a Phase I study for molecular imaging of acute venous thromboembolism. The trial was registered with the identifier: NCT02864810. Another study employed FLECT (fluorescence emission computed tomography) to target the same receptors (GPIIb/IIIa), providing an optical imaging modality to generate a 3D prediction of thrombosis 61. Additionally, a theranostic approach by Wang et al. combined activated platelet targeting with the delivery of a fibrinolytic drug and they demonstrated accurate imaging combined with a high thrombolytic efficacy without any bleeding complications 142. While PET imaging provides a relatively high sensitivity, its application specifically in cardiovascular diseases is problematic. This is due to the combination of both the long image acquisition times of PET and the complex motion patterns of coronary arteries, which continuously move with each heartbeat. Therefore, in regard of motion artifacts, other imaging modalities such as CT might be more suitable 143. Another difficulty in atherosclerosis imaging is that, although the symptoms evolve very slowly, the ongoing inflammation process is very dynamic resulting in differential expression of target molecules 144. With this in mind, the long circulation time of full length IgG antibodies might give rise to higher background signals and irrelevant results. To this end, Broisat et al. screened 10 different nanobodies to select a lead candidate that has maintained nanomolar affinity while possessing a rapid blood clearance as well (which is favorable in this case). They demonstrated the suitability of nanobodies in targeting VCAM-1 and thereby image vulnerable atherosclerotic plaques 145. A more recent study in 2018 employed PET/MRI imaging of atherosclerosis in rabbits while testing nanobodies against 3 different molecular targets: VCAM-1, LOX-1 and MMR^47^. We believe that the complexity of the inflammatory process in atherosclerotic plaques requires multiple imaging probes in order to make an accurate assessment of plaque stability. Furthermore, multimodal imaging is necessary to combine sensitivity with penetration depth while correcting for motion artifacts.

To conclude, the recent development in molecular imaging holds great potential in the field of cardiovascular diseases. Individuals develop atherosclerosis over a period of decades without the presence of any clinical symptoms 146. An early, non-invasive diagnostic tool can establish routine screenings of people selected based on their risk profile and determine the presence or absence of cardiovascular abnormalities. This way, an intervention either therapeutically or non-therapeutically (e.g. lifestyle change) might prevent future fatal cardiovascular injury.

## Conclusion

This review covered the application of monoclonal antibodies in a variety of molecular imaging modalities. Three major applications were highlighted (oncology, autoimmune disease and cardiovascular disease) to demonstrate how universally applicable molecular imaging is. This universality relies on the high affinity, high specificity and wide repertoire that monoclonal antibodies offer. While antibodies are popular in bio-medical research, they can still be improved using genetic engineering to generate a variety of fragments or fusion protein with different properties. Each application demands its own tailor-made probe and solely relying on native, full-length monoclonal antibodies limits the full potential that antibody engineering has to offer. The combination of therapy and diagnostics, theranostics, prompted much attention in recent years due to its high potential in personalized treatments and disease management. The one-size-fits all approach slowly becomes out of fashion as biologics offer new tools to tailor each treatment to each patient and to each state of the disease. The latter is especially relevant to molecular imaging as the state of the disease changes constantly and with it the expression of certain markers/targets. A routinized theranostic modality that continuously monitors the state of the disease (e.g. in terms of receptor expression) and adjusts the therapy accordingly, holds great potential for the future of precision medicine.

Despite the overwhelming amount of research in small animals, clinical translation of molecular imaging modalities is limited, especially in more complex multimodal imaging. While nuclear imaging has been the leading technology in medical imaging for many years, new alternatives are explored, as nuclear probes possess some drawbacks. Clinical facilities require sufficient budget and should be in close proximity to a cyclotron in order to perform routine imaging of patients. The short half-life of radionuclides limits the timeframe and this already present complexity is getting more complicated when conjugating radionuclides to proteins.

Radionuclide-protein conjugates add an extra layer of complexity, as they are not only limited by half-life but also require specific conditions to remain stable (pH, temperature, salt concentration etc.). Furthermore, the intolerance of proteins to chemical modifications requires novel technologies to readily attach proteins to radionuclides without compromising functionality. Some examples of these were described in this review and provide innovative solutions to such limitations (e.g. adding reactive groups during protein translation by using genetic code expansions). However, the implementation of such innovative ideas from small animal imaging to clinical studies takes time. With the development of fluorescence emission computed tomography (FLECT) or fluorescence molecular tomography (FMT), the field of optical imaging got a boost. These, fluorescence-based optical imaging modalities utilize fluorophores (mainly in the near-infrared region) which are less difficult to manufacture than radionuclides and remain stable for a longer period.

Another limitation of the translation of molecular imaging to the clinic is the higher complexity in image analysis as compared to classical medical imaging. To exploit the full potential of (multimodal) molecular imaging, a multidisciplinary team of physicians/scientists is required to understand the biological process underlying the medical image. However, new developments in machine learning and big data can overcome these hurdles. We believe that the development of new imaging modalities and novel conjugation approaches combined with machine learning programs that use big data to learn to detect abnormalities in patients will drive the transition to clinical applications.

In an ideal scenario, the future of molecular imaging involves repertoires of molecular probes that are tested not only in patients but also in healthy individuals to allow for early disease detection by routine screening. These molecular probes should employ multimodal imaging to take benefits of each available imaging modality. Computer programs that learned to detect even the smallest abnormalities analyze the enormous amounts of generated 3D images and reveal physicians with unprecedented accuracy what the state of the disease is.

## Figures and Tables

**Table 1:** An overview of commonly used imaging modalities, its properties, (dis)advantages and imaging agents used to enable molecular imaging approaches.

Imaging modality	Imaging agent for targeting	Properties	Advantages	Disadvantages	Examples
NUCLEAR IMAGING					
Positron emission tomography (PET)	Shorter-lived radionuclides such as ^64^Cu, ^18^F and ^15^O 50	Based on positron emitting radionuclides. Measures high-energy photons produced during annihilation of the positron upon interaction with an electron. Creates 3D images.	Superb sensitivity, unlimited penetration depth. Highly developed as molecular imaging modality.	Short half-life radionuclide requires fast procedure and nearby cyclotron. No anatomical information. Low spatial resolution. Expensive equipment.	MYC and transferrin receptor targeted imaging of triple negative breast cancer^51^, imaging of Alzheimer's disease by targeting CatD^52^
Single-photon emission computed tomography (SPECT)	Longer-lived Radionuclides such as ^111^In, ^99m^Tc and ^123^I 50	Based on the detection of gamma ray emissions by radionuclide decay. Creates 3D images.	Longer decay half-lives than PET and therefore more widely available. Less expensive. Possibility to employ multiple radionuclides in parallel.	Less sensitive than PET. Like PET it has a low spatial resolution and provides no anatomical information.	Monitoring effect of anti-angiogenic therapy in lung cancer^53^, SPECT/CT imaging of glioma using targeted gold nanoparticles^54^
Scintigraphy	Similar to SPECT	Similar to SPECT, detects gamma rays upon radionuclide decay. However, in contrast to SPECT, scintigraphy generates 2D images.	Longer decay half-lives than PET and therefore more available. Less expensive.	Less sensitive than PET, no anatomical information and only 2D images.	Identification of HER2+ tumors using ^111^In/labeled Trastuzumab^55^,
OPTICAL IMAGING					
Photoacoustic imaging (PAI)	Absorbing small-molecule dyes, metallic nanoparticles 15	Detection of low-amplitude ultrasound waves generated by localized thermo-elastic expansion in tissue upon adsorption of pulsed laser light.	No ionizing radiation involved, cost-effective and portable imaging devices.	Limited penetration depth, shorter wavelengths result in weak absorption, temperature dependent.	Detection of prostate cancer by targeting PSMA^56^, Imaging tumor vascular permeability with indocyanine green^57^
Bioluminescence imaging (BLI)	Luciferase expressing gene 58	Detection of light produced by the enzymatic reaction of luciferase and its substrate	Higher sensitivity and lower background than fluorescence imaging	Substrate required.	Imaging caspase-3 activity with luciferase expression after doxorubicin treatment of tumors^59^
Fluorescence molecular tomography (FMT)	Small synthetic fluorescent probes or fluorescent protein-expressing genes 60	Optical imaging that employs fluoroprobes for optical tomography.	Accurate quantification in deep tissue. Generates a 3D image. No ionizing radiation involved.	Background signals caused by autofluorescence. Requires transillumination of animals in contrast to surface illumination in epifluorescent approaches.	Near infrared imaging of activated platelets^61^, Imaging of glioblastoma in mice^62^
OTHER IMAGING MODALITIES					
Magnetic resonance imaging (MRI)	Superparamagnetic iron oxide (SPIO), gadolinium-DTPA 58	Based on NMR in the presence of a magnetic field. ^1^H is often used as a nuclide. Differentiation between different tissues is based on difference in behavior of water molecules in tissues under a magnetic field.	Employs non-ionizing radiation. More detailed images than CT in soft tissues.	Low sensitivity: necessitates longer acquisition times and more imaging agent material. Noise might cause hearing issues.	Comparison of several molecular probes in MRI imaging of breast cancer patients^63^, VCAM-1 targeted MRI for early detection of brain micrometastases^64^
Computed tomography (CT)	Nanoparticle-based contrast agents. Contrast agents are relatively new, CT used to be purely anatomical 65	Based on differences in X-ray absorption among different tissues. X-ray source and detector rotate around the subject in order to generate cross-sectional images from which a 3D image is computed.	Deep tissue penetration, high spatial resolution and fast generation of 3D image	No functional information as a CT scan is rather anatomical (although this is changing since recently). High dose of ionizing radiation.	Targeted CT imaging of cervical cancer using gold nanoparticles^66^, Anti-CD24 as a targeted contrast agent in CT imaging of cancer cells^67^
Ultrasound (US)	Contrast agents such as microbubbles or nanoparticles^68^	Acoustic waves compress e.g. microbubbles with the positive pressure and expand it with the negative pressure. This creates asymmetric echoes that can be measured.	Good temporal resolution, relatively inexpensive, no ionizing radiation.	Contrast agents are large and don't diffuse easily into tissue	Imaging of angiogenesis in tumors using VEGFR-2-targeted microbubbles 69
